# Ovarian steroid cell tumors, not otherwise specified: analysis of nine cases with a literature review

**DOI:** 10.1186/s12902-022-01170-9

**Published:** 2022-11-01

**Authors:** Mengyan Lin, Kechun Bao, Lingjia Lu, Shuhang Xu, Yun Liang, Xiaodong Cheng, Fenfen Wang

**Affiliations:** 1 Department of Gynecological Oncology, Women’s Hospital, Zhejiang University School of Medicine, No.1 Xueshi Road, 310006 Hangzhou, Zhejiang Province China; 2 Department of Pathology, Women’s Hospital, Zhejiang University School of Medicine, Hangzhou, China

**Keywords:** Ovarian steroid cell tumors, Not otherwise specified, Treatment

## Abstract

**Background:**

Ovarian steroid cell tumors (SCTs), not otherwise specified (NOS), are rare, with few large studies. The purpose of this study was to analyze the clinical features, prognosis, and treatment choices for these patients of different age groups.

**Methods:**

This was a retrospective study. We identified nine cases of ovarian steroid cell tumor, not otherwise specified, confirmed by post-operative histopathological examination, and analyzed clinical features, surgical procedures, and follow up outcomes. We also reviewed cases reports of ovarian steroid cell tumors, not otherwise specified.

**Results:**

A total of nine cases were included. The age range was 9–68 years (mean, 41.89 ± 19.72 years). Clinical features included virilization, amenorrhea, abdominal pain, vaginal bleeding, isosexual precocious puberty, Cushing’s syndrome, and abnormal weight gain with elevated testosterone levels. The follow up interval ranged 5–53 months and no recurrence was observed.

**Conclusion:**

Ovarian steroid cell tumors covered all age groups, with manifestations of androgen excess. Younger patients appeared to have a more favorable prognosis, which provided more opportunities for these patients to pursue treatment options that will preserve reproductive function.

## Introduction

Ovarian cancers are subdivided into epithelial and non-epithelial counterparts. Non-epithelial ovarian cancers are rare, accounting for approximately 10% of all ovarian cancers and include mainly germ cell tumors, sex cord-stromal tumors, and some extremely rare tumors [1]. Ovarian steroid cell tumors (SCTs) are rare ovarian sex cord stromal tumors that account for less than 0.1% of all ovarian tumors [2]. SCTs occur in females of all ages, causing symptoms such as virilization, isosexual precocious puberty in adolescents, Cushing’s syndrome, and infertility. These clinical manifestations may result from excessive secretion of steroid hormones as well as tumor mass effects. There are three subtypes of SCT, according to cell origin: stromal luteomas, Leydig cell tumors, and not otherwise specified (NOS) tumors [2]. Approximately 60% of SCTs are of the NOS subtype and are usually benign; however, some of these tumors do have malignant behavior, as is observed with peritoneal metastases [3, 4]. Living qualities and fertility are partially associated with the clinical manifestations of SCT-NOS as well as the different treatment options. Here, we describe the clinical characteristics of nine cases of SCT-NOS and review literature reports related to this disease. The aim of this study was to summarize features for diagnosis, treatment, and prognosis of ovarian SCT-NOS and provide new clues for future treatment.

We present the following article in accordance with the STROBE reporting checklist.

## Methods

We included all cases diagnosed as ovarian SCT-NOS through histopathological examinations in the Women’s Hospital, Zhejiang University School of Medicine from January 2013 to December 2020. There were 12 retrospective cases, and three of these were excluded because there was a lack of patient history, history of operations at other centers, or because patients were lost to follow up. The remaining nine cases included patients who had received surgical treatment. Clinical records, biological results, histological results, and follow-up information were analyzed for these patients.

## Results

### Clinical features

For the nine cases analyzed, patient age ranged from 9 to 68 years (mean, 41.89 ± 19.72 years). One patient was premenarchal, four were of reproductive age, and four were postmenopausal. Adnexal masses were the reason for operation in six cases; the other SCTs were incidentally discovered after operations related to endometrial cancers (two cases) or endometriosis (one case).

Most of the nine ovarian SCT patients experienced symptoms of endocrine or metabolic disorders. Three patients presented with virilization symptoms such as hirsutism, acne, and hoarseness; virilization symptoms were alleviated in all patients within 2–3 months. Amenorrhea was observed in two patients along with virilization symptoms. In one patient, regular menses resumed 2 months post-operation, while amenorrhea persisted in the other patient due to administration of gonadotropin releasing hormone agonist (GnRHa) therapy. Two patients reported abdominal pain and one experienced irregular vaginal bleeding. Isosexual precocious puberty presenting as early development of breasts, occurred in one case involving an 9-year-old girl, and was alleviated postoperatively. Five patients were overweight [body mass index (BMI) ≥ 24 kg/m^2^] and two were obese (BMI ≥ 28 kg/m^2^); Cushing’s syndrome was observed in one obese patient (Table 1).

**Table 1:** Clinical features of nine patients

No	Age (years)	Clinical manifestations					BMI (kg/m^2^)	T (nmol/L)	DHEAS (µmol/L)
		**Lower abdominal mass**	**Abdominal discomfort**	**Endocrine disorders**	**Obesity/ overweight**	**Others**			
1	14	+	Abdominal pain	Amenorrhea, acne, hirsutism, Cu-shing’s syndrome	Obesity	-	30.11	3.8↑	9.68↑
2	53	+	-	-	-	-	23.83	6.1↑	6.78
3	25	+	-	Amenorrhea, hirsutism, acne,	Obesity	Fatty liver	29.64	17.6↑	8.25↑
4	55	-	-	Irregular vaginal bleeding	Overweight	Endometrial cancer stage IIIC	24.09	1.4	8.34↑
5	51	+	-	Hirsutism	Overweight	-	27.97	/	/
6	59	+	-	-	-	-	19.43	5.2↑	11.8↑
7	43	+	-	-	-	Dysmenorrhea, endometriosis	22.20	0.4	5.2
8	68	-	-	Regular vaginal bleeding until 65-years-old	Overweight	Endometrial cancer stage IA	24.75	0.9	6.1
9	9	+	Abdominal pain	Premature mammary development	-	-	13.72	0.8	/

Clinical symptoms typically differ based on hormonal secretion, and preoperative serum androgen levels were elevated in most patients. Five patients had abnormal hormone levels. In four patients, serum testosterone (T) levels were increased to 3.8–17.6 nmol/L (normal range, 0.3–3.0 nmol/L). In four patients, preoperative serum dehydroepiandrosterone-sulfate (DHEAS) levels were elevated to 8.25–11.8 µmol/L (normal range, 0.96–6.95 µmol/L). Testosterone and DHEAS levels returned to normal postoperatively in two patients (Table 1). One menopausal patient had slightly higher estradiol as well as slightly lower follicle-stimulating hormone (FSH) and luteinizing hormone (LH) levels (FSH: 34.98 IU/L, LH: 21.89 IU/L, estradiol: 101.9 pmol/L), according to normal menopausal levels (FSH: >40 IU/L, LH: >25 IU/L, estradiol: <100 pmol/L).

Pelvic masses were found in seven patients. Pelvic ultrasound revealed a heterogeneous mass in five patients, along with lower blood flow resistance index (RI, range 0.31–0.52); one patient exhibited no obvious blood flow signal. Magnetic resonance imaging (MRI) of five patients indicated a T1 isointense and T2 hyperintense mass. The lesions were well enhanced after administration of gadolinium-diethylenetriamine pentaacetic acid. Although pelvic masses were present in most patients, one patient did not present with an ovarian mass preoperatively and was ultimately diagnosed by pathology.

### Surgical treatment

Four patients underwent bilateral salpingo-oophorectomy with or without hysterectomy, and three young patients underwent ovarian tumor resection. Other procedures occurred in only one patient, as described in Table 2. Two cases were diagnosed with SCT-NOS based on histopathological diagnosis without visible ovarian tumors. Tumor diameter ranged from 2 to 5 cm in the other six cases (Table 2). Macroscopic examination revealed yellowish-brown, soft, solid masses in four cases. One case showed a mixture of cystic and solid dark red masses and the other showed a hard, solid mass. Thus, a pelvic mass was a typical sign of SCT-NOS, and surgical resection was an important treatment.

**Fig. 1:**
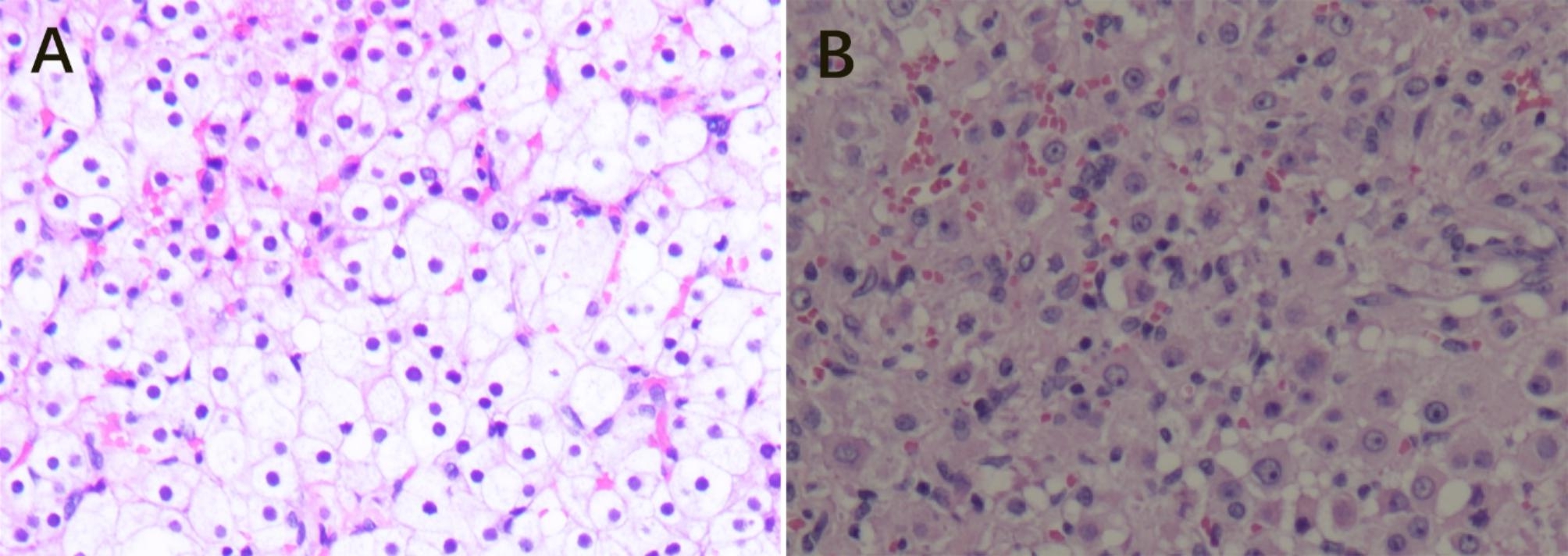
Microscopic appearance of steroid cell tumor, not otherwise specified (H&E). (**A**) Benign SCT-NOS cells with abundant cytoplasm (20×). (**B**) SCT-NOS cells with nuclear atypia grade II–III with abundant cytoplasm (20×)

Histopathological features of eight cases are described in Table 2 (Fig. 1). Hemorrhage and nuclear atypia grade II–III were discovered in a 68-year-old patient with endometrial cancer. Nuclear atypia grade II–III was observed alone in a 9-year-old patient. In these cases, neither necrosis nor two or more mitotic cells per 10 high power fields were found.

### Follow up

Follow-up time for the nine cases ranged from 5 to 53 months. Disease recurrence had not occurred in any of the cases recently (Table 2).

**Table 2:** Surgical procedures and follow-up of nine patients

No	Age (years)	Surgical procedure	Tumor size (≥ 7 cm)	Pathological features				Follow-up (months)	Recurrence
				**Necrosis**	**Hemorrhage**	**Mitosis (≥ 2 per 10 HPF)**	**Nuclear atypia (grade II/III)**		
1	14	OTR	-	-	-	-	-	5	No
2	53	RSO	-	-	-	-	-	9	No
3	25	OTR	-	-	-	-	-	11	No
4	55	HE, BSO, OE, PLND	-	-	-	-	-	12	No
5	51	HE, BSO	/	/	/	/	/	27	No
6	59	BSO	-	-	-	-	-	28	No
7	43	HE, BS, OER	-	-	-	-	-	32	No
8	68	EHE, BSO	-	-	+	-	+	46	No
9	9	OTR	-	-	-	-	+	53	No

OTR: ovarian tumor resection; RSO: right salpingo-oophorectomy; BSO: bilateral salpingo-oophorectomy; BS: bilateral salpingectomy; HE: hysterectomy; EHE: extrafascial hysterectomy; OE: omentectomy; PLND: pelvic lymph node dissection; OER: ovarian endometriotic cyst resection

## Discussion

**Fig. 2:**
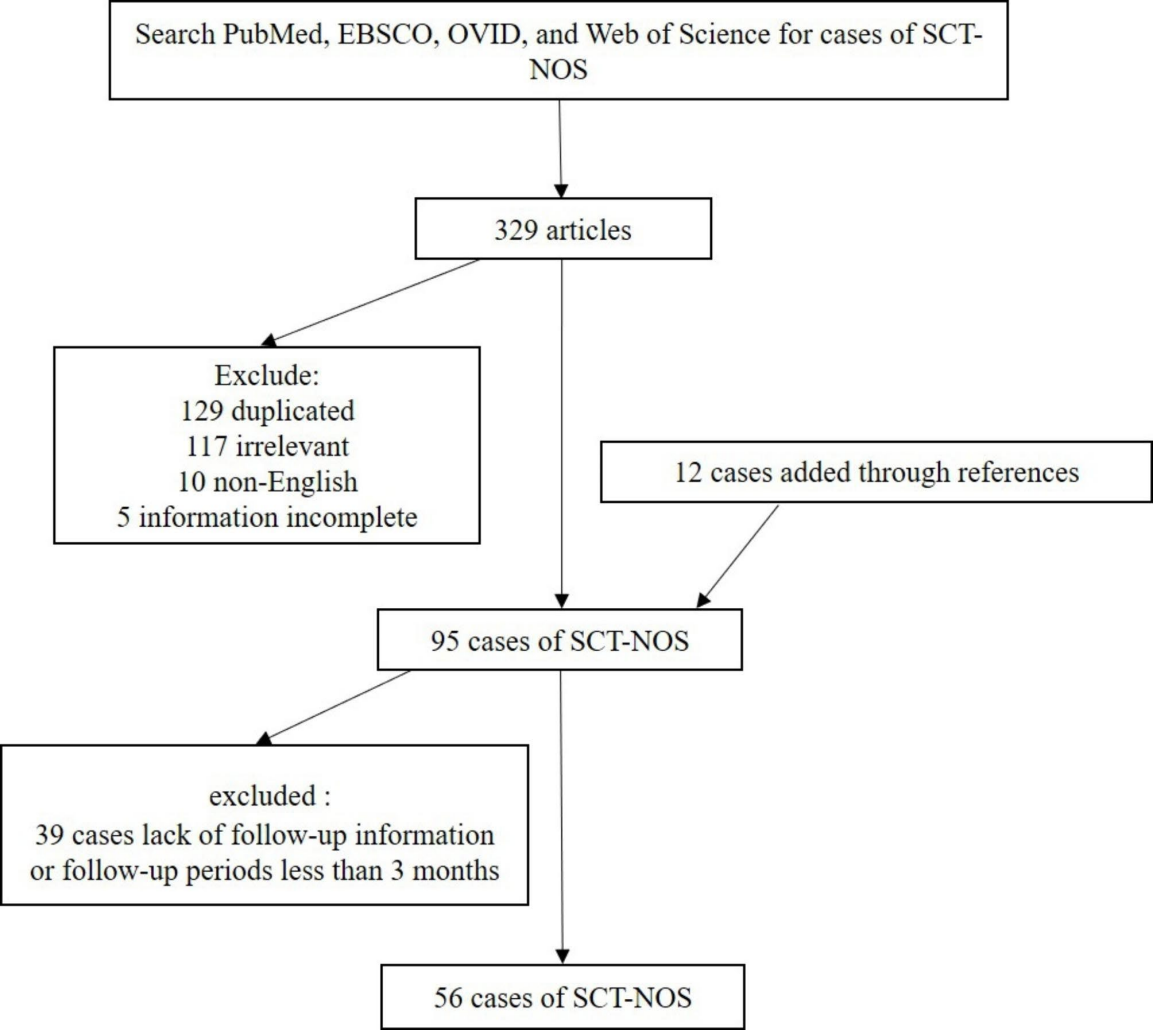
Workflow diagram for the search

Ovarian SCT-NOS have not been studied across a large number of cases, except for Hayes and Scully’s investigation of 63 cases [2]. We searched PubMed, EBSCO, OVID, and Web of Science for cases of SCT-NOS in English with the search string ((“steroid cell tumor” OR “steroid cell neoplasm”) AND (ovary OR ovaries OR ovarian)) AND (“not otherwise specified” OR “nos”). A total of 329 articles were found, of which 129 were duplicates, 117 were irrelevant, 10 were non-English, and five contained incomplete information. These unmatched articles were excluded and 12 other case reports were added through references. Ultimately, we found 80 articles describing 95 cases of SCT-NOS. We excluded 39 of these cases due to lack of follow-up information or follow-up periods that were too short (less than 3 months), which left 56 cases (Table 3; Fig. 2). We did not include the information from the 63 cases in Hayes and Scully’s article because details were lacking in each of these cases.

**Table 3:** Clinical features of 65 cases reported

Case	First author	Age (years)	Surgical procedure	Tumor diameter (cm)	Pathological features				Adjuvant therapy	Follow-up (months)
					**Necrosis**	**Hemorrhage**	**Mitosis (≥ 2 per 10 HPF)**	**Nuclear atypia (grade II/III)**		
1	Haroon [14]	3	LSO	7	-	-	-	-	-	No recurrence (25)
2	Yoshimatsu [15]	4	RSO	8	+	+	+ (3)	-	BEP×3	No recurrence (7)
3	Qian [16]	5	RSO	8	-	-	-	-	-	No recurrence (60)
4	Gupta [17]	5	Tumor resection	NA	NA	NA	NA	NA	-	No recurrence (4)
5	Sawathiparnich [18]	6	LSO	7	+	-	+ (2–5)	-	-	No recurrence (6)
6	Lee [19]	8	RO	5	-	-	-	-	-	No recurrence (16)
7	Bas [7]	9	HE, BSO	2.5	-	-	-	-	Glucocorticoid	No recurrence (52)
8	Yilmaz [5]	13	Tumor resection	2.5	-	-	-	-	-	No recurrence (6)
9	Schnuckle [20]	14	LSO	3.2	-	+	-	-	-	No recurrence (24)
10	Yokozawa [6]	16	Tumor resection	4	-	-	-	-	-	No recurrence (15)
11	Yuan [21]	17	Tumor resection	8	-	-	-	-	-	No recurrence (6)
12	Sielert [22]	20	RO	5.5	-	-	-	-	-	No recurrence (18)
13	Jiang [23]	21	HE, BSO, OE	L 20, R 15	+	+	+ (>10)	+	PVB×4	Continued and died (10)
14	Santo [24]	21	RSO	11.5	-	+	+ (4)	-	-	No recurrence (43)
15	Zhang [25]	21	LSO, OE	5.8	-	-	-	-	-	No recurrence (3)
16	Matsukawa [26]	22	Tumor resection	3	-	-	-	-	-	No recurrence (60)
17	Arora [27]	22	HE, LSO	2	NA	NA	NA	NA	-	Recurred (108)
18	Jiang [23]	23	Tumor resection	6	-	-	-	-	-	No recurrence (36)
19	Wong [28]	24	RSO	4.5	-	-	-	-	-	No recurrence (9)
20	Haroon [14]	26	RSO	4.5	-	-	-	-	-	No recurrence (15)
21	Menghua [29]	28	LO	2.5	+	NA	Active cell growth	NA	-	Recurred (42), died (58)
22	Swain [30]	28	LSO	5.9	NA	NA	NA	NA	-	No recurrence (12)
23	Haroon [14]	28	HE, BSO	3 & 2.5	-	-	+ (2)	-	-	No recurrence (49)
24	Li [31]	29	RSO	6	+	+	+	+	Docetaxel + Ne-daplatin ×8	Recurred (18), died (24)
25	Tai [8]	29	RSO	10	-	+	-	-	-	No recurrence (24)
26	Alves [4]	30	Tumor resection	2	-	-	-	-	-	No recurrence (36)
27	Liu [32]	30	Tumor resection	3	-	-	-	-	-	No recurrence (12)
28	Haroon [14]	32	HE, BSO	4	-	-	-	-	-	No recurrence (71)
29	Chung [33]	35	HE, BSO, PLND, OE, AE	4.9	-	-	-	-	GnRHa ×6	No recurrence (43)
30	Stephens [34]	35	RSO	5	NA	NA	-	NA	-	No recurrence (6)
31	Faten [9]	36	RO	4.5	-	-	-	-	-	No recurrence (12)
32	Haroon [14]	37	LSO	7	-	-	-	-	-	No recurrence (23)
33	Ben [35]	39	LSO	3.4	NA	NA	NA	NA	-	No recurrence (8)
34	Chen [36]	40	RSO	6	NA	NA	NA	NA	-	No recurrence (36)
35	Varras [37]	40	HE, BSO	6.8	-	-	-	-	-	No recurrence (18)
36	Haroon [14]	43	RSO	10	-	-	+ (5)	+	-	Recurred (34)
37	Zang [38]	46	LSO	12	NA	NA	NA	-	-	No recurrence (12)
38	Kim [39]	46	HE, BSO, PLND, OE	7.7	NA	NA	NA	NA	-	Recurred (60)
39	Reedy [40]	46	HE, LSO, PLNS, OE	3.6	-	-	-	-	-	No recurrence (12)
40	Lee [41]	47	HE, BSO, OE	11	-	+	+ (5)	+	BEP ×3	No recurrence (24)
41	Wang [42]	48	HE, RSO, PLND, OE	4	-	-	-	+	-	No recurrence (6)
42	Nakasone [43]	50	HE, BSO, PLNS	9	-	-	NA	-	-	Recurred (70)
43	Haroon [14]	50	RSO	7	-	-	-	-	-	No recurrence (11)
44	Haroon [14]	51	HE, BSO, PLND, OE	13	+	+	+ (8)	+	-	Recurred and died (8)
45	Chun [44]	52	LSO	6	-	-	+ (5)	-	-	No recurrence (21)
46	Kim [45]	52	BSO, PLNS, OE	7.5	-	-	-	-	-	No recurrence (24)
47	Haroon [14]	52	LSO	9	-	-	+ (4)	+	PVB ×4 & radioth-erapy	No recurrence (43)
48	Cooray [46]	54	HE, BSO	L 3, R 4	-	-	-	-	-	No recurrence (12)
49	Faraj [47]	55	BO	4.5	NA	NA	NA	NA	-	No recurrence (12)
50	Layla [48]	65	BSO, TD	7	-	NA	+ (2)	-	BEP ×6	Continued (18)
51	Brewer [49]	58	HE, BSO, TD	10	NA	NA	+ (9)	+	BEP ×2, GnRHa	Recurred (28)
52	Sedhom [50]	67	HE, BSO, TD	9.8	NA	+	+ (22)	NA	-	Died after operation
53	Stephens [51]	67	HE, BSO	1.2	-	-	-	-	-	No recurrence (3)
54	Haroon [14]	67	BSO	3.4	-	-	-	-	-	No recurrence (27)
55	Singh [52]	70	RO	5	NA	NA	NA	NA	-	No recurrence (5)
56	Powell [53]	93	HE, BSO, sigmoid-ectomy, colostomy	21	+	NA	+	+	-	Recurred and multi metastasis (10)

LSO: left salpingo-oophorectomy; RSO: right salpingo-oophorectomy; BSO: bilateral salpingo-oophorectomy; LO: left oophorectomy; RO: right oophorectomy; BO: bilateral oophorectomy; HE: hysterectomy; OE: omentectomy; PLND: pelvic lymph node dissection; PLNS: pelvic lymph node sampling; AE: appendectomy; TD: tumor debulking; NA: not available; BEP: bleomycin + etoposide + cisplatin; PVB: cisplatin + vincristine + bleomycin

Of the 56 cases, the age span of SCT-NOS patients was 3–93 years (median, 33.5 years). Excessive weight gain and virilization features such as hirsutism, acne, baldness, hoarseness, clitoromegaly, along with elevated testosterone were common at any age. For patients of reproductive age, amenorrhea was persistent because of hormonal parasecretion. For premenarchal girls, isosexual precocious puberty and advanced growth affected most. In our cases, virilization manifestations such as hirsutism, acne, and amenorrhea, as well as excessive weight gain were also quite common due to testosterone elevation. Isosexual precocious puberty occurred in one nine-year-old patient from our set of cases, and presented as premature mammary development. Other less common manifestations such as hypertension, abdominal pain, vaginal bleeding, Cushing’s syndrome, and acanthosis nigricans were also reported in the cases analyzed in this study. The symptoms caused by SCT-NOS vanished within 1–16 months post-operation, among the cases with no recurrence. However, Yilmaz and Yokozawa discovered that some changes, including hoarseness, were irreversible even after hormone secretion abnormalities were corrected by tumor elimination [5, 6]. Whether grown adolescent’s height and weight were normalized could not be determined due to lack of follow-up data. A previous study by Bas reported that a 9-year-old SCT-NOS patient had coexistent congenital adrenal hyperplasia due to classic 11 beta-hydroxylase deficiency, and the patient had initial hypertension that progressed and showed hypertensive retinopathy 2 years post-operation [7]. Alves, Faten, and Tai each published cases involving infertility with follow-up periods of 36 months, 12 months, and 24 months, respectively; none of the cases reported successful pregnancy [4, 8, 9]. Data related to infertility were limited due to lack of long-term follow-up. The reported pregnancy rate of non-epithelial ovarian cancer after fertility sparing surgeries varied from 50 to 93%, and live-birth rate was 65-95% [10]. Akimasa and Öz published two gestational SCT-NOS cases with full-term delivery through cesarean, along with unilateral salpingo-oophorectomy and the tumor resection, respectively; both two cases underwent a second staging procedure [11, 12]. The reported rate of fetus preservation was 69.4% for gestational patients of ovarian sex-cord stromal tumors who underwent fertility sparing surgeries in the second and third trimesters, and 60.9% patients delivered at term [13]. Some menopausal cases exhibited elevated estradiol as well as low LH and FSH levels, which may have induced endometrial disease from persistent estradiol influence. In our cases, two postmenopausal patients also presented with endometrial adenocarcinoma. Although reproductive hormone levels corresponded with postmenopausal levels in both patients, one also experienced vaginal bleeding until age 65 year. It is suspicious that long-term, persistent estradiol stimulation resulted in vaginal bleeding symptoms, and this suggests the potential presence of STC-NOS along with induced endometrial adenocarcinoma.

Approximately 25–43% SCTs-NOS have malignant potential [2]. The previous study by Hayes and Scully pointed out five pathological features that were correlated with malignancy potential: (1) two or more mitotic fields per 10 high power fields (92% malignant); (2) necrosis (86% malignant); (3) tumor diameter ≥ 7 cm (78% malignant); (4) hemorrhage (77% malignant); and (5) grade II–III nuclear atypia (64% malignant) [2]. Among the 56 cases searched, with the exception of eight cases with missing pathological details, approximately half (28/48) presented with at least one malignant feature. Metastasis was observed in seven cases with primary operation, and at least two malignant pathological features were reported in six cases. Another case was reported according to only one feature. Two other cases showed capsule and vascular infiltration or vascular growth, respectively, and both presented with at least two features. In patients 40 years or younger, the rate of malignant pathological features was 44.83% (13/29), and the rate for patients over 40 years of age was 78.95% (15/19) (P˂0.05, χ2 test). Metastasis, infiltration, and vascular growth occurred in 10.34% (3/29) of the patients 40 years or younger and in 31.58% (6/19) of the patients over 40 (P˃0.05, χ2 test). It seems that SCTs-NOS in patients over the age of 40 are more likely to have malignant behavior. In our cases, a 68-year-old patient experienced hemorrhage and nuclear atypia grade II–III, and a 9-year-old patient presented with nuclear atypia grade II–III. The sample size was limited in this study, and larger scale clinical studies comparing different ages are still needed to provide more conclusive evidence.

**Fig. 3:**
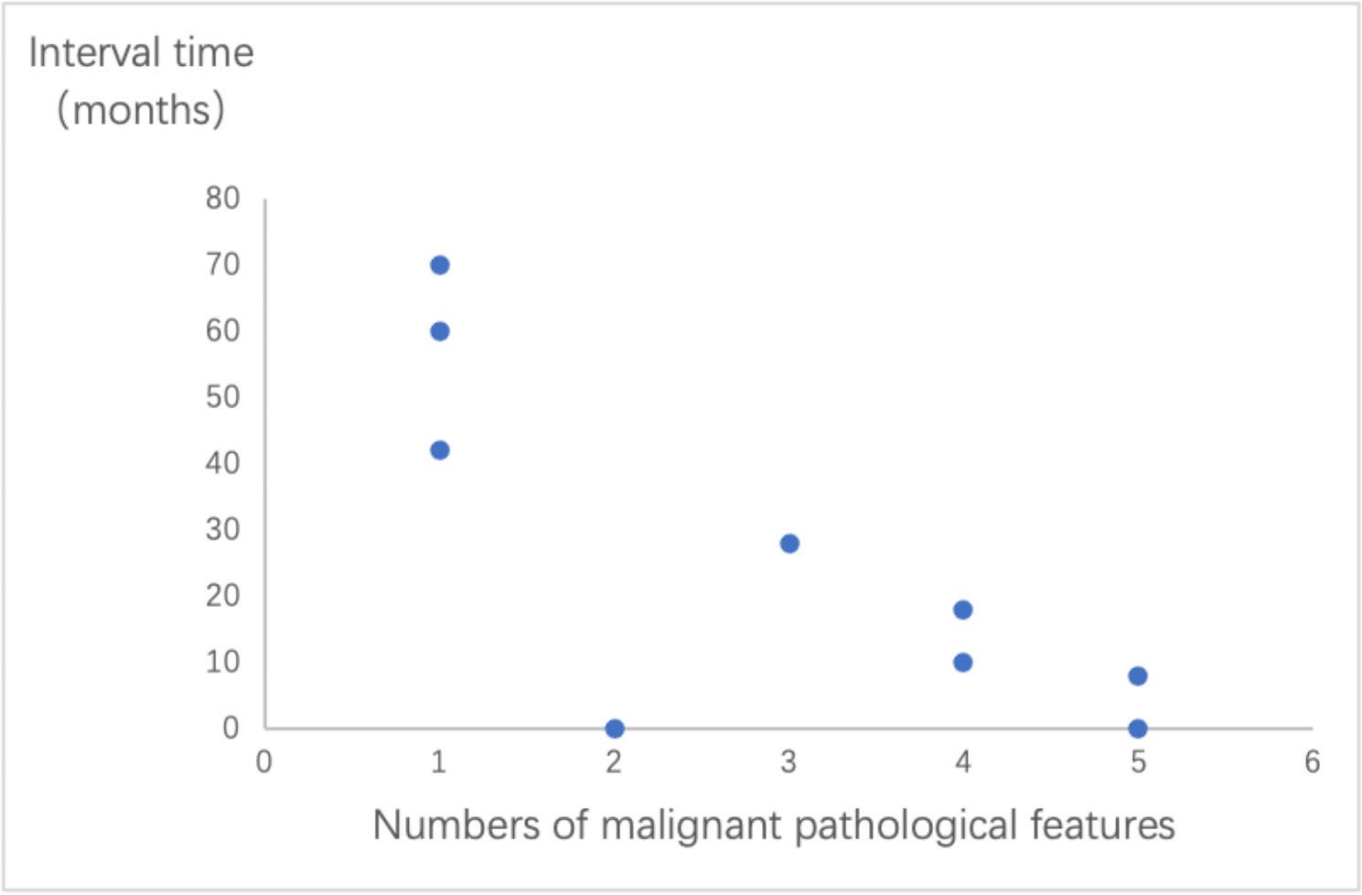
Relationship between number of malignant pathological features and interval time

Disease recurrence or progression occurred in 10 cases (17.86%) (Table 4). The tumor-free interval ranged from 0 to 108 months, with a median time of 23 months. The age of recurrence ranged from 21 to 93 years, and none of patients younger than 20 years of age reported disease recurrence or progression. The recurrence rate was 11.43% (4/35) for patients aged 40 years or younger, 28.57% (6/21) for those older than 40 years. In the group above 40 years of age, malignant behavior and disease recurrence were more likely to happen. Ascites was a unique manifestation for this group and may serve as an indicator of recurrence following initial therapy. One case that recurred did not report any malignant pathological features, with the longest tumor-free interval of 9 years. Six cases displayed at least two malignant pathological features, whereas three cases displayed only one. The tumor-free interval increased considerably as the feature numbers decreased (Fig. 3).

**Table 4:** Clinical features, treatments, and outcomes of recurred or progressed cases

Case	Age (years)	Clinical features	Second therapy	Outcome (months)†
13	21	Virilization, amenorrhea, ascites, acanthosis nigricans, initial surgery delayed 4 years	PVB×4	Continued and died (10)
17	22	Virilization, amenorrhea, with hereditary leiomyomatosis and renal cell carcinoma syndrome	RSO, RPLND	Not mentioned
21	28	Virilization, Cushing’s syndrome, hypertension, irregular menstruation	Metastatic lesions resection	Recurred (12), died (16)
24	29	Lower back and leg pain, normal reproductive hormone	Not mentioned	Died (6)
38	46	Ascites	Metastatic lesions resection, BEP	Tumor free (9)
42	50	Virilization	BEP×3, TP×8, chemotherapy invalid, GnRHa	No symptom (22 after first GnRHa)
44	51	Lower abdominal pain, vaginal spotting, anorexia	Not mentioned	Died
51	58	Virilization, pelvic discomfort	Increased dose of GnRHa	Not mentioned
50	65	Virilization, abdominal pain, ascites, bilateral lower limb edema	BEP×3	Continued (18), lost to follow up
56	93	Virilization, abdominal pain, bilateral lower limb edema, aggressive and violent behavior	GnRHa & weekly paclitaxel for 1 month	Refused therapies and progressed

†The interval time after recurrence or progress; PVB: cisplatin + vincristine + bleomycin; BEP: bleomycin + etoposide + cisplatin; TP: paclitaxel + carboplatin; RSO: right salpingo-oophorectomy; RPLND: right pelvic lymph node dissection

The reported treatments for disease recurrence or progression in SCT-NOS were tumor excision operations, chemotherapy, and GnRHa therapy. Two recurrent cases received surgery alone, with one reporting 12 months tumor free and a survival time of 16 months. In two reported cases, tumor progression showed no response to chemotherapy alone. Another relapsed case did not respond to chemotherapy but recovered after shifting to GnRHa therapy, and remained tumor free for 22 months. This suggested that for relapsed SCT-NOS patients, GnRHa therapy may be an appropriate choice for adjuvant treatment. Brewer reported a case with aggravated symptoms as well as computed tomography (CT) showing enlarged lymph nodes and uronephrosis during chemotherapy alone after the primary operation; however, the symptoms faded, lymph nodes were diminished, and uronephrosis was improved after switching from chemotherapy to GnRHa [49]. This again indicated an important role for GnRHa as an SCT-NOS adjuvant treatment.

Tumor resections alone were performed in eight patients aged 40 years or younger with no reported disease recurrence. Unilateral salpingo-oophorectomies or oophorectomies were performed in 20 patients aged 40 years or younger, and there were two reports of disease recurrence. These surgeries helped to preserve ovarian function and fertility for young patients with an occurrence rate of 7.14%, which was lower than the overall rate. As discussed above, the malignancy potential and occurrence appeared to be lower in people younger than 40 years and, therefore, surgeries that preserve fertility and ovarian function can be considered when obvious malignant features are absent.

## Conclusion

Due to the low incidence of the SCT-NOS, it was difficult to conduct large-scale clinical cohort studies and there was a lack of consistent recommendations for treatment. SCT-NOS are a rare ovarian tumor subtype that can occur at any age. Virilization, weight gain, and amenorrhea with elevated testosterone secretion are common among SCT-NOS patients. Isosexual precocious puberty can also occur in immature girls. Though endocrine abnormalities often recover after surgery, the resulting development effects remain. Therefore, it is important to improve the early diagnosis of this disease to reduce abnormal development caused by endocrine factors as well as relieve anxiety and psychological pressure in younger patients. For older patients, SCT-NOS has malignant potential, and it is important to properly complete pathological assessment because the risk of malignancy and the recurrence rate both increase after age of 40 years. It is of the upmost importance for clinicians to pay careful attention to this phenomenon and effectively manage it. Younger patients have a more favorable prognosis, which provides the chance to consider fertility and ovarian function preserving surgeries.

This study included mostly cases published in public journals, with necessary complete information and prognostic outcomes, between 1991 and 2020. This relatively complete retrospective clinical study provides effective information and value for the diagnosis and treatment of SCT-NOS.

## Data Availability

The datasets used and analysed during the current study are available from the corresponding author on reasonable request.

## Abbreviations

SCT: ovarian steroid cell tumor.
NOS: not otherwise specified.
GnRHa: gonadotropin releasing hormone agonist.
BMI: body mass index.
T: testosterone.
DHEAS: dehydroepiandrosterone-sulfate.
FSH: follicle-stimulating hormone.
LH: luteinizing hormone.
RI: resistance index.
MRI: magnetic resonance imaging.
OTR: ovarian tumor resection.
RSO: right salpingo-oophorectomy.
LSO: left salpingo-oophorectomy.
BSO: bilateral salpingo-oophorectomy.
BS: bilateral salpingectomy.
HE: hysterectomy.
EHE: extrafascial hysterectomy.
OE: omentectomy.
PLND: pelvic lymph node dissection.
RPLND: right pelvic lymph node dissection.
PLNS: pelvic lymph node sampling.
OER: ovarian endometriotic cyst resection.
LO: left oophorectomy.
RO: right oophorectomy.
BO: bilateral oophorectomy.
AE: appendectomy.
TD: tumor debulking.
NA: not available.
BEP: bleomycin + etoposide + cisplatin.
PVB: cisplatin + vincristine + bleomycin.
TP: paclitaxel + carboplatin.
CT: computed tomography.
